# Long-term remission and biologic persistence rates: 12-year real-world data

**DOI:** 10.1186/s13075-020-02380-z

**Published:** 2021-01-13

**Authors:** Kieran Murray, Matthew Turk, Yousef Alammari, Francis Young, Phil Gallagher, Tajvur Saber, Ursula Fearon, Douglas J. Veale

**Affiliations:** 1 Department of Rheumatology, St Vincent’s University Hospital, Dublin 4, Ireland; 2 EULAR Centre for Arthritis and Rheumatic Diseases, Dublin, Ireland; 3 Lady Reading Hospital, Soekarno Rd, PTCL Colony Peshawar, Khyber Pakhtunkhwa, 25000 Pakistan; 4 Molecular Rheumatology, School of Medicine, Trinity Biomedical Sciences Institute, Trinity College Dublin, Dublin, D06 R590 Ireland

**Keywords:** Rheumatoid arthritis, Psoriatic arthritis, Biologics, Remission

## Abstract

**Background:**

Biologic therapies have greatly improved outcomes in rheumatoid arthritis (RA) and psoriatic arthritis (PsA). Yet, our ability to predict long-term remission and persistence or continuation of therapy remains limited. This study explores predictors of remission and persistence of the initial biologic therapy in patients after 12 years. Furthermore, outcomes with adalimumab and etanercept are compared.

**Patients and methods:**

RA and PsA patients were prospectively recruited from a biologic clinic. Outcomes on commencing therapy, at 1 year and 12 years were reviewed. Demographics, medications, morning stiffness, patient global health score, tender and swollen joint counts, antibody status, CRP and HAQ were collected. Outcomes at 1 year and 12 years are reported and predictors of biologic persistence and EULAR-defined remission (DAS28-CRP < 2.6) are examined with univariate and multivariate analysis.

**Results:**

A total of 403 patients (274 RA and 129 PsA) were analysed. PsA patients were more likely to be male, in full-time employment and have completed higher education. PsA had higher remission rates than RA at both 1 year (60.3% versus 34.5%, *p* < 0.001) and 12 years (91.3% versus 60.6%, *p* < 0.001). This difference persisted when patients were matched for baseline disease activity (*p* < 0.001). Biologic continuation rates were high for RA and PsA at 1 year (49.6% versus 58.9%) and 12 years (38.2% versus 52.3%). In PsA, patients starting on etanercept had lower CRP at 12 years (*p* = 0.041). Multivariate analysis showed 1-year continuation [OR 4.28 (1.28–14.38)] and 1-year low-disease activity [OR 3.90 (95% CI 1.05–14.53)] was predictive of a 12-year persistence. Persistence with initial biologic at 12 years [OR 4.98 (95% CI 1.83–13.56)] and male gender [OR 4.48 (95% CI 1.25–16.01)] predicted 12 year remission.

**Conclusions:**

This is the first study to show better response to biologic therapy in PsA compared to RA at 12 years. Long-term persistence with initial biologic agent was high and was predicted by biologic persistence and low-disease activity at 1 year. Interestingly, PsA patients had higher levels of employment, educational attainment, and long-term remission rates compared to RA patients.

## Background

RA and PsA are the most common forms of inflammatory arthritis (IA) with a prevalence of 1% and 0.25%, respectively [1, 2]. They are chronic relapsing/remitting multisystem conditions characterised by synovitis, joint deformity, loss of function and increased mortality [3–6]. The economic implications for patients, their families and society are significant [7]; RA has an estimated annual economic burden of over €45 billion in Europe alone [8].

Biologic disease modifying anti-rheumatic drugs (bDMARDs) have dramatically improved outcomes in RA, PsA, psoriasis and other autoimmune conditions [9]. They are superior to methotrexate in decreasing disease activity [10, 11], and they improve physical function in conventional synthetic DMARD (csDMARD) failures and inhibit radiographic damage [12]. Tumour necrosis factor inhibitors (TNFis) reduce mortality and are more cardioprotective than csDMARD treatment [13, 14]. Compared to methotrexate, bDMARDs are cost-effective first-line therapies due to greater efficacy, fewer dose-limiting adverse effects and lower monitoring costs [15]. The current treatment paradigm has shifted to bDMARD as first-line therapies [9]. Indeed, updated ACR guidelines advise a TNFi over traditional csDMARDs in treatment-naive patients with active PsA [16].

Numerous RCTs examine bDMARDs in IA [17–21]. However, these may not be generalizable to routine clinical practice due to strict inclusion criteria, short duration and relatively small sample sizes [22]. Registries can provide more real-world data [23–30]. Again, these may only follow patients for a limited time and, in comparison to RA, there remains a paucity of data on biologic use in PsA [24]. Given the lack of RCTs comparing TNFi, observational studies are required [31]. In this prospective study, we compare outcomes with adalimumab and etanercept. Despite new bDMARDs, these two agents continue to account for 61% of biologic prescriptions by Irish rheumatologists [32].

A meta-analysis found rates of 1 year biologic persistence, defined as a continuation of a therapy, in RA varied from 32.0 to 90.9% [33]. Less is known about a long-term biologic persistence in PsA [34]. Among German patients on subcutaneous biologics, a higher proportion of PsA patients (57.9%) remained on therapy ≥ 12 months compared to RA (51.9%) [35]. In the US Corrona registry, 36.1% of PsA patients continued on their medication at 48 months. Medication continuation studies are often based on administrative data, lacking important clinical and laboratory parameters [36].

This study compares clinical, laboratory and epidemiological characteristics of a well characterised real-world cohort of RA and PsA patients commencing bDMARDs. Many studies examining predictors of biologic continuation and remission are limited to 1 year. We examined for longer term predictors by providing 12-year outcomes and biologic continuation data. Furthermore, we compare disease activity, remission and medication continuation for the two most commonly prescribed subcutaneous bDMARDs—adalimumab and etanercept. We provide insights into patient characteristics that predict long-term disease course and biologic continuation for individual patients.

## Methods

### Study design

In 2000, a specific biologic outpatient clinic and prospective database was created to enable close monitoring of these novel therapies. This clinic is described in detail in a prior study in this journal [37]. This current study compares outcomes on commencing therapy, at 1 year and most recent review.

Baseline, 1-year (1.04 ± 0.15 years) and 12-year (11.76 ± 2.69 years) assessments were compared. Demographics (age, gender, educational level, employment), smoking status, diagnosis, disease duration, medications, Health Assessment Questionnaire (HAQ), patient global health (PGH), tender joint count (TJC), swollen joint count (SJC), C-reactive protein (CRP), rheumatoid factor (RF) and anti-cyclic citrullinated peptide antibody (ACPA) status and erosive status on X-rays of the hands and feet were reviewed. Erosive status was defined by the presence or absence of erosions on standard plain X-rays of the hands and feet as reported by a consultant musculoskeletal radiologist. The 28-joint count Disease Activity Score with CRP (DAS28-CRP) was calculated, as it is a validated measure in both RA and PsA clinical trials, and remission was classified as DAS28-CRP < 2.6 according to the European League Against Rheumatism (EULAR) criteria [38, 39]. Disease activity was defined as low (DAS28-CRP ≤ 3.2), moderate (3.2 < DAS28-CRP ≤ 5.1) or high (DAS28-CRP > 5.1) [38, 40, 41]. Patients who did not commence a named biologic at the clinic review were excluded from the study (*n* = 64).

### Statistical analysis

Data analyses were performed using SPSS 26. Nominal data is presented as frequencies and percentages. Normally distributed continuous and ordinal data are presented as the mean and standard deviation (SD), while non-normally distributed data are presented as the median (range). Between group differences were analysed using unpaired 2-tailed *t* tests, Pearson chi-square, Fisher’s exact test or Mann-Whitney *U* test as appropriate.

Univariate analysis was used to identify predictors of (i) persistence with initial biologic agent and (ii) remission at 12 years. All factors that demonstrated a significant association within the univariate models (*p* < 0.20) were then used to create a multivariate model and evaluated with logistic regression analysis. Age and education were retained in the multivariate models even if not significant as is standard practice for these biologically important confounders. Odds ratios (ORs) from these two final models quantified the effect of each factor as a predictor of remission or biologic continuation.

## Results

### Patient characteristics

A total of 403 patients (274 RA and 129 PsA) were followed-up. RA patients tended to be older, more commonly female and had more severe disease with higher HAQ scores and more erosions (Table 1). Patients with PsA had higher educational attainment and employment levels (Table 1). To check for confounders explaining the difference in educational levels, a subset of male patients 40–59 years old were analysed. The difference in educational level maintained statistical significance. 52.4% of patients with PsA were university graduates versus 19.2% in RA (*p* = 0.014). In this subgroup, there was no significant difference in employment (*p* = 0.261).

**Table 1:** Baseline characteristics

	RA (*n* = 274)	PsA (*n* = 129)	***p***
**Age**, years	55 ± 11.9	44.8 ± 12.2	< 0.001
**Female**	207 (75.5%)	69 (53.5%)	< 0.001
**Disease duration,** years	9 (0–51)	8 (0–40)	0.030
**Education level completed**			
Primary School	246 (100%)	98 (100%)	NS
Secondary School	176 (71.5%)	90 (91.8%)	< 0.001
University	56 (22.8%)	59 (60.2%)	< 0.001
**Employment**			
Employed	37 (66.1%)	31 (91.2%)	0.008
Unemployed	8 (14.3%)	1 (2.9%)	NS
Student	0	1 (2.9%)	NS
Other	11 (19.6%)	1 (2.9%)	0.027
**Hours worked**, weekly	37.2 (11.7)	41.1 (9.1)	NS
**Smoking status**			
Never	89 (35.6%)	50 (45.5%)	NS
Ex	94 (37.6%)	35 (31.8%)	
Current	67 (26.8%)	25 (22.7%)	
**HAQ score**	1.3 (0.64)	0.89 (0.64)	< 0.001
**EMS**, minutes	30 (0–1440)	30 (0–1440)	NS
**RF positive**	195 (77.1%)	2 (2.2%)	< 0.001
**ACPA positive**	31 (79.5%)	0 (0%)	< 0.001
Erosions*	90 (47.1%)	28 (31.5%)	0.013

Values are expressed as *n* (%), mean (SD) or median (range)

*HAQ* Health Assessment Questionnaire, *EMS* early morning stiffness, *RF* rheumatoid factor, *ACPA* Anti-citrullinated protein antibodies

^*^On X-rays of the hands and feet

### Medications

**Fig. 1:**
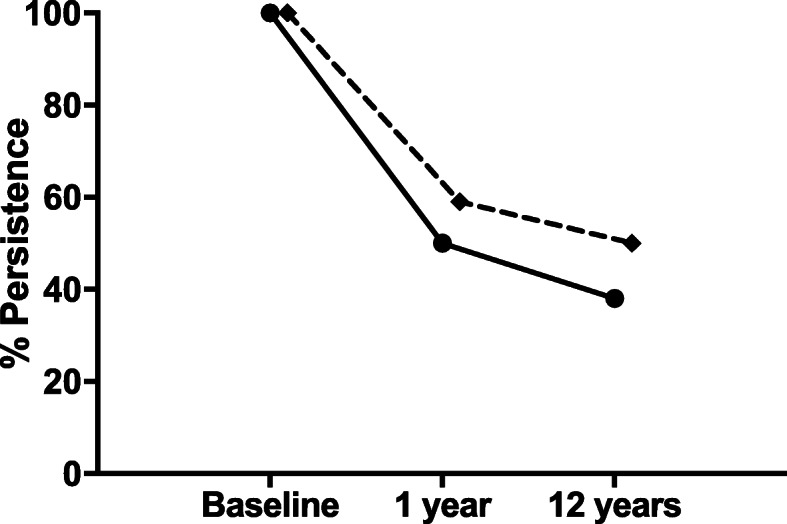
A comparison of persistence with initial biologic agent over time in rheumatoid and psoriatic arthritis. Persistence rates with original biologic agent in rheumatoid arthritis is represented by the solid line (*n* = 274) and psoriatic arthritis (*n* = 129) by the dotted line

Patient medications are shown in Table 2. At baseline review, the majority of RA patients commenced adalimumab, with most PsA patients starting etanercept. A small number of patients in both groups received infliximab and 4.4% of RA patients started on rituximab. Figure 1 shows the rate of persistence with the original bDMARD. There was no significant difference between RA and PsA at 1 year (*p* = 0.088) or 12 years (*p* = 0.068). At 12 years, 20.2% of RA and 11.9% of PsA patients were no longer on a bDMARD and 14.3% of RA and 9.5% of PsA patients were prescribed a novel bDMARD/small molecule inhibitor (Table 2). Methotrexate use was higher in RA at all three time points (Table 2).

**Table 2:** A comparison of rheumatoid arthritis and psoriatic arthritis outcomes at biologic initiation, one year and 12 year reviews

	Baseline			1 Year Review			12 Year Review		
	RA (*n* = 274)	PsA (*n* = 129)	*p*	RA (*n* = 203)	PsA (*n* = 96)	*p*	RA (*n* = 179)	PsA (*n* = 87)	*p*
**Biologic**									
Adalimumab	144 (52.6%)	47 (36.4%)	0.002	76 (44.7%)	27 (31.0%)	0.034	43 (25.6%)	28 (33.3%)	NS
Etanercept	100 (36.5%)	68 (52.7%)	0.002	62 (36.5%)	45 (51.7%)	0.019	48 (28.6%)	30 (35.7%)	NS
Infliximab	18 (6.6%)	14 (10.9%)	NS	13 (7.6%)	11 (12.6%)	NS	3 (1.8%)	8 (9.5%)	0.004
Rituximab	12 (4.4%)	0	0.011	4 (2.4%)	0	NS	16 (9.5%)	0	0.004
Other^a^	0	0		1 (0.6%)	0	NS	24 (14.3%)	8 (9.5%)	NS
None	0	0		14 (5.1%)	4 (3.1%)	NS	34 (20.2%)	10 (11.9%)	NS
**Biologic persistence**	n/a	n/a	n/a	136 (49.6%)	76 (58.9%)	NS	66 (38.2%)	45 (52.3%)	NS
**Methotrexate**	176 (64.2%)	35 (27.3%)	<0.001	108 (64.8%)	21 (24.4%)	<0.001	72 (42.2%)	22 (25.9%)	0.008
**PGH**, mm	60 (0-100)	50 (0-100)	NS	30 (0-100)	20 (0-90)	<0.001	50 (0-100)	5 (0-100)	<0.001
**TJC28**	9 (0-28)	6 (0-28)	<0.001	1 (0-28)	0 (0-20)	<0.001	0 (0-19)	0 (0-2)	0.019
**SJC28**	9 (0-28)	5 (0-28)	<0.001	1 (0-28)	0 (0-25)	<0.001	0 (0-15)	0 (0-2)	<0.001
**CRP**, mg/L	16 (2-158)	9 (0-108)	<0.001	4 (1-138)	4 (0-41)	0.034	3 (1-65)	2 (1-46)	<0.001
**Disease Activity**									
Low	9 (3.5%)	13 (11.1%)	0.003	91 (55.2%)	60 (82.2%)	<0.001	83 (79.8%)	22 (95.7%)	NS
Moderate	89 (34.4%)	65 (55.6%)	<0.001	58 (35.2%)	10 (13.7%)	0.001	20 (19.2%)	1 (4.3%)	0.012
High	160 (61.8%)	39 (33.3%)	<0.001	16 (9.7%)	3 (4.1%)	NS	1 (1%)	0 (0%)	NS
**Erosion progression**				36 (28.1%)	5 (8.6%)	0.003	51 (31.9%)	12 (15.0%)	0.005

PGH…Patient Global Health Visual Analogue Score (mm), TJC28..28-joint tender joint count, SJ28…28-joint swollen joint count ^a^Alternative biologic or small molecule inhibitor-golimumab, certolizumab, tocilizumab, abatacept, secukinumab, ustekinumab, ixekizumab tofacitinib or baricitinib

### Clinical response

**Fig. 2:**
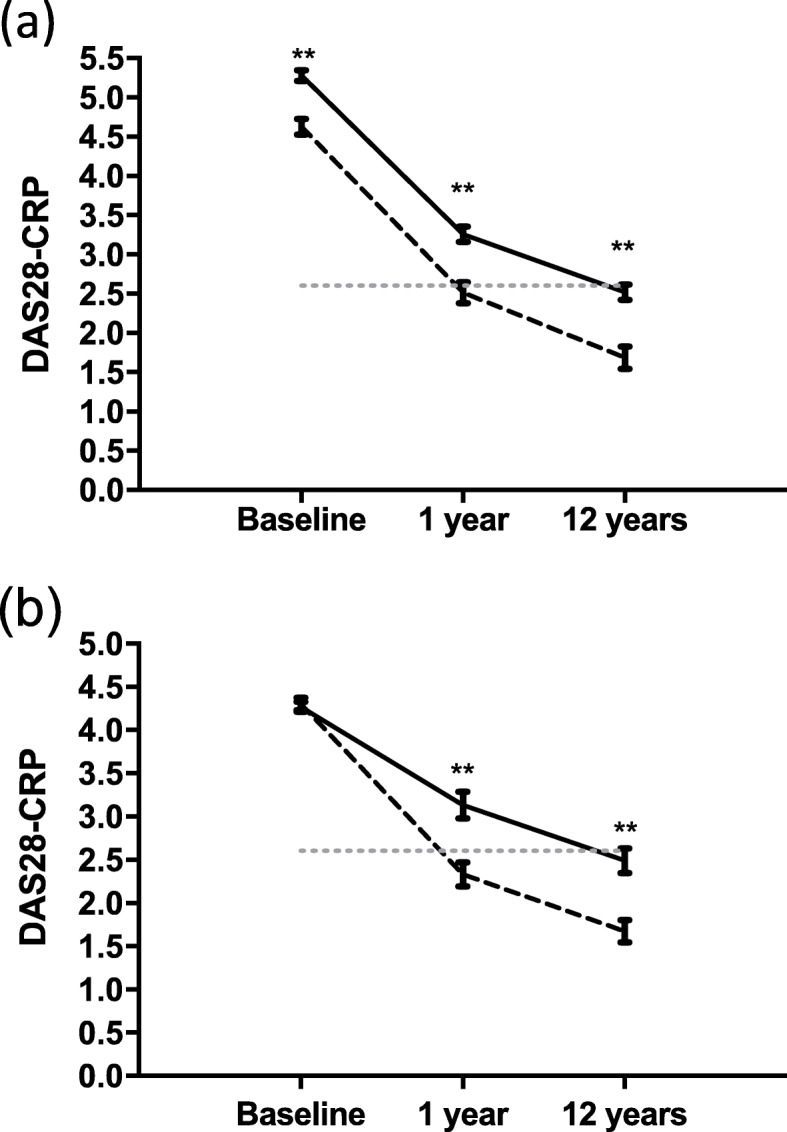
A comparison of DAS28-CRP in RA and PsA across 12 years. **a** Line graph representing DAS28-CRP responses to biologic therapy at 1 year and 12 years in RA [solid line (*n* = 274)] and PsA [dotted black line (*n* = 129)]. Remission is represented by the dotted grey line at DAS28-CRP value of 2.6. DAS28-CRP is significantly lower in PsA at all time points. **b** Line graph representing DAS28-CRP responses to biologic therapy at 1 year and 12 years in a cohort matched for baseline DAS28-CRP (moderate disease activity) in RA [solid line (*n* = 89)] and PsA [dotted black line (*n* = 65)]. Remission is represented by the dotted grey line at DAS28-CRP value of 2.6. Values are expressed as mean +/− SEM; **p < 0.001 between RA and PsA at that timepoint

**Fig. 3:**
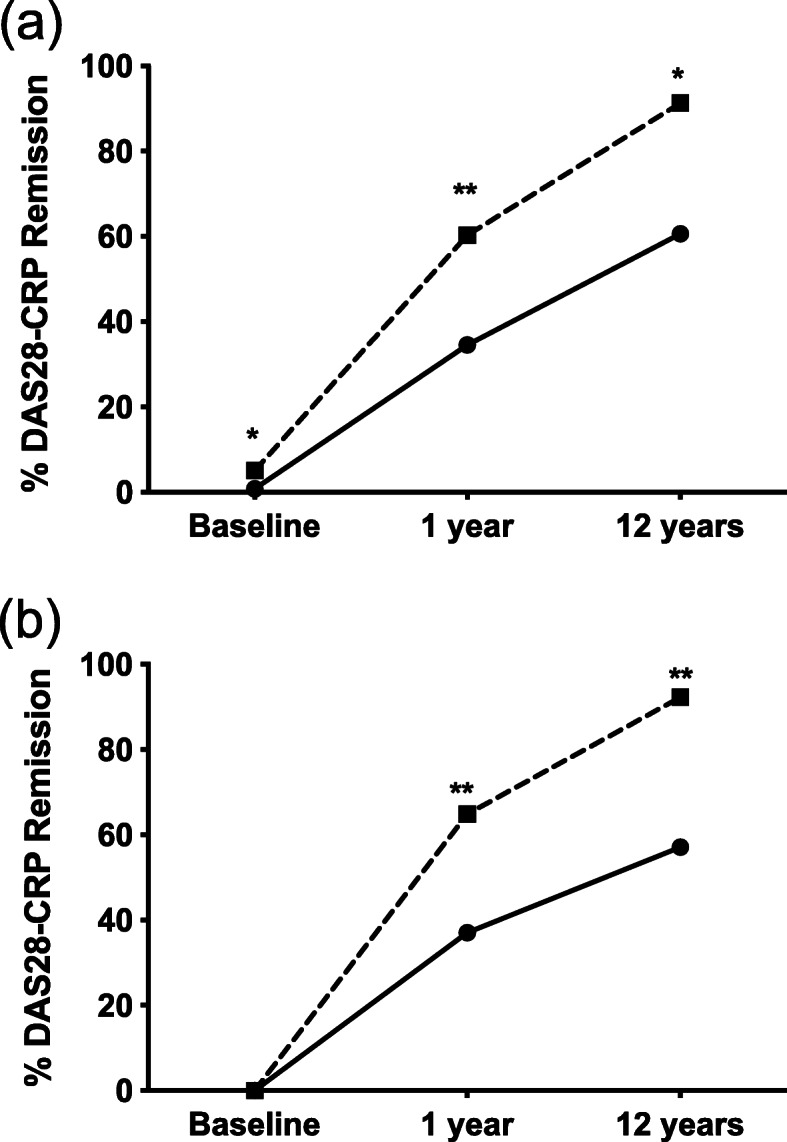
A comparison of rates of DAS28-CRP remission over time. **a** PsA represented by the dotted line (*n* = 129) has higher remission rates at all time points when compared to RA (*n* = 274), represented by solid black line. **b** In patients matched for baseline disease activity [moderate disease activity (*n* = 154)], remission rates are higher in PsA at both 1 year and 12 years *p < 0.05, **p < 0.001 between RA and PsA at that timepoint

Table 2 shows the dramatic improvement in clinical outcomes in both diseases. Median TJC improved from nine to zero in RA and six to zero in PsA. SJC improved from nine to zero in RA and five to zero in RA and PsA, respectively. CRP was higher in RA, as expected, at all three points. The rate of progression of erosions was also higher among patients with RA at both 1 year (28.1% versus 8.6%, *p* = 0.003) and 12 years (31.9% versus 15.0%, *p* = 0.005). DAS28-CRP was higher in RA at all three time points (Fig. 2a). One year and 12-year DAS28-CRP remained higher in RA, even in a cohort of patients matched for disease activity at baseline (moderate disease activity) (Fig. 2b). Remission rates were higher in PsA at all three timepoints (Fig. 3a). Even in the subgroup matched for baseline DAS-28 CRP, both 1 year and 12-year remission rates remained higher in PsA (Fig. 3b).

### Predictors of continuation and remission

The multivariate analysis focused on predictors of a 12-year continuation with initial bDMARD and a 12-year remission. One year continuation [OR 4.28 (1.28–14.38)] and 1-year low-disease activity [OR 3.90 (95% CI 1.05–14.53)] predicted continuation (Table 3). A 12-year continuation [OR 4.98 (95% CI 1.83–13.56)] and male gender [OR 4.48 (95% CI 1.25–16.01)] predicted remission (Table 4).

**Table 3:** Univariate and multivariate analyses for predictors of persistence with initial biologic at 12 years

	Univariate	Multivariate	
	***p***	OR (95% CI)	***p***
**Persistence at 1 year**	0.011	4.28 (1.28–14.38)	0.019
**One-year low-disease activity**	< 0.001	3.90 (1.05–14.53)	0.042
**Smoker/ex-smoker at baseline**	0.055	0.39 (0.14–1.10)	0.074
**Completed university**	0.033	1.84 (0.54–6.27)	0.333
**Etanercept at baseline**	0.020	1.51 (0.56–4.07)	0.411
**Baseline alcohol use**	0.135	1.37 (0.47–4.04)	0.565
**Infliximab at baseline**	0.156	0.50 (0.05–5.00)	0.557
**Male gender**	0.061	0.75 (0.24–2.38)	0.623
**Finished secondary school**	0.010	0.85 (0.26–2.78)	0.784
**Remission at 1 year**	0.002	0.87 (0.27–2.86)	0.823
**Baseline methotrexate use**	0.084	1.07 (0.40–2.89)	0.897
**Age > 50 years**	0.797	0.97 (0.32–2.90)	0.950

**Table 4:** Univariate and multivariate analyses for predictors of a 12-year remission

	Univariate	Multivariate	
	***p***	OR (95% CI)	***p***
**12-year biologic persistence**	0.002	4.98 (1.83–13.55)	0.002
**Male gender**	0.003	4.48 (1.25–16.01)	0.021
**University graduate**	0.057	2.49 (0.68–9.09)	0.166
**1-year remission**	0.017	2.01 (0.71–5.71)	0.190
**Age > 50 years**	0.127	1.15 (0.37–3.61)	0.813

### Comparative analysis of subcutaneous bDMARDs

In this study, 89.1% (*n* = 359) of patients were on one of the two subcutaneous bDMARDs, etanercept and adalimumab. In comparison to etanercept, patients on adalimumab were more likely to be older (53.7 ± 12.4 years versus 49.4 ± 13.0 years, *p* = 0.001) and have RA [144 (75.4%) versus 100 (59.5%), *p* = 0.001]. When separated by disease, patients on each agent were well matched (Supplementary Tables 1 and 2). There were no significant differences in rates of continuation with initial bDMARD or methotrexate use in either disease (Supplementary Table 3 and 4). Clinical response was excellent with both agents. There were no significant differences between agents in RA at any timepoint (Supplementary Table 3). In PsA, patients on etanercept had a higher baseline DAS28-CRP (mean 4.95 ± 1.10) than those on adalimumab (mean 4.45 ± 0.93) (*p* = 0.011) (Supplementary Table 4). At 12 years, PsA patients initially commenced on etanercept had a slightly lower CRP (median 2.1 mg/L, range 1–31) compared with adalimumab (median 1.1 mg/L, range 1–46) (Supplementary Table 4).

## Discussion

This is the first study to show better 12 year bDMARD response in PsA compared to RA. Overall, the patients have excellent outcomes, with 91.3% of PsA and 60.6% of RA patients in remission at 12 years. This compares favourably to other long-term studies [42–44]. Progress of erosions was also less frequent [45]. Despite lower levels of methotrexate use, disease activity was lower in PsA based on all outcome metrics at all time points except for baseline patient global health (PGH). Interestingly, PGH has been shown to be a confounder in both diseases [46–48]. This study delivers some clear messages; PsA is generally a milder clinical phenotype than RA and patients with PsA respond better to bDMARD therapies. In comparative analysis of subcutaneous bDMARDs, outcomes were very similar for adalimumab and etanercept in both RA and PsA.

Consistent with prior studies, the RA patients tended to be older, more commonly female and had more severe disease (with higher HAQ scores and more erosions) [37]. With respect to demographics, patients with PsA had higher educational attainment and employment levels. In a subgroup matched for age and gender, levels of university education remained higher in patients with PsA. This is the first study to show differences in educational attainment between RA and PsA. The prevalence of IA is increased among patients with psoriasis, ranging from 7 to 42%, compared with a general population estimate of 2 to 3% [49]. In about 85%, psoriasis precedes articular involvement [4]. Perhaps, better educated individuals with psoriasis may be more aware of their increased risk of PsA and thus present to medical attention more promptly. A previous study in our unit showed PsA patients with low education status were significantly more likely to have a diagnostic delay of > 2 years (OR 2.09, *p* = 0.02) [50]. In that study, a 6-month delay from symptom onset to the first visit with a rheumatologist contributes to the development of peripheral joint erosions and worse long-term physical function [50]. Late presentation is also a poor prognostic factor in RA [51, 52].

Demographics can be predictive of outcomes in IA. This study and many others show males have better outcomes and response to bDMARDs in IA [53, 54]. A Danish study showed a 2-fold lower risk of RA among those with the longest formal education compared with those having the lowest level of education [55]. An American study found educational level to be an important marker of RA clinical status [56]. Individuals who did not complete high school had worse outcomes according to ESR, joint count, grip strength and walking time [56]. In our study, the unemployment rate in RA was 14.3%, compared to the national unemployment rate of 4.3%. In the current study, 28.5% of RA patients did not complete secondary school. Patient education is important in medication adherence, and patient information should be delivered at a 13–14-year-old reading level [57]. However, we have previously shown much of the health information available for arthritis is above this level [57]. It is important to recognise social determinants of health when we interact with patients in clinic in order to optimise their outcomes.

In RA, mean DAS28-CRP fell from 5.28 ± 1.10 at baseline to 2.51 ± 0.99 at 12 years (*p* < 0.001). In PsA, the mean baseline DAS28-CRP was 4.60 ± 1.11 and fell to 1.68 ± 0.69 after 12 years (*p* < 0.001). Even when matched for disease activity at baseline, PsA had a lower mean DAS28-CRP compared to RA at 1 year and 12 years suggesting a better response to bDMARD therapy.

Many prior studies examining predictors of biologic persistence and remission are limited to 1 year. In contrast, we examined for predictors after 12 years of treatment. Our multivariate analyses highlight the difficulty in predicting long-term continuation and remission at bDMARD commencement. In the multivariate model, no baseline factors were predictive of continuation with initial bDMARD. Male gender, however, was predictive of remission. Our observation that male gender is associated with a greater rate of remission is consistent with prior studies [42, 53, 58, 59]. The underlying mechanism for this relationship remains poorly understood. Male sex hormones seem protective in IA. Men with RA tend to have higher levels serum of oestrodiol levels and lower levels of androgenic steroids including testosterone and dehydroepiandrosterone compared to healthy controls [60, 61]. TNF blockade affects hormone levels in the synovial fluid prior to serum levels and seems to block the aromatase induced conversion of androgens to oestrogens [62].

One-year bDMARD persistence and 1-year low-disease activity were significant predictors of long-term bDMARD continuation. This is useful information in clinical practice. Continuation with initial bDMARD and quiescent disease at 1 year is predictive of longer term outcomes. A long-term bDMARD continuation was predictive of remission. Interestingly, methotrexate use is low, particularly at the 12 year review (42.2% in RA and 25.9% in PsA). Methotrexate is the most widely used first-line RA therapy [63, 64]*.* Methotrexate is therapeutically effective, cost-effective and, unlike other csDMARDs, has a mortality benefit in RA (hazard ratio 0.4 (95% CI 0.2–0.8) [65, 66]. The addition of methotrexate to bDMARD therapy has been shown to decrease disease activity, slow radiographic progression and improve function in a RCT [67]. However, methotrexate has significant limitations. It may take 6 months to achieve full therapeutic response [18, 68, 69]. Despite widespread methotrexate use in PsA, we lack randomised placebo-controlled trials showing a decrease in radiographic progression or benefits sustained beyond 6 months [70]. In one study of methotrexate monotherapy in PsA, < 20% of patients achieved minimal disease activity at 6 months [71]. Adverse effects include nausea, headaches, cytopenia, hepatotoxicity, pulmonary toxicity and teratogenicity [72–75]. Blood monitoring is burdensome. Current guidelines suggest taking full blood count, liver function tests and urea and electrolytes on 7 separate occasions within the first 18 weeks of therapy [76]. Some guidelines also advise alcohol abstinence [77]. Given the difficulties associated with use and the high remission rates in our cohort it is, perhaps, not surprising methotrexate use is low.

One- and 12-year persistence rates were 49.6% and 38.2% in RA and 58.9% and 52.3% in PsA, respectively. Numerous studies show a higher bDMARD continuation in spondyloarthropathies such as PsA when compared to RA [78, 79]. A 2014 meta-analysis of > 200,000 RA patients on bDMARD therapy found continuation rates of 73% at 1 year and 48% at 4 years [80]. In the BSR register, 69.2% of PsA patients were persistent with initial bDMARD at 2.3 years [81]. An Italian registry study found bDMARD continuation levels of 85% at 1 year and 64% at 3 years [78].

In the current study, both of the subcutaneous bDMARDs (etanercept and adalimumab) commenced at baseline showed excellent clinical outcomes. There were no differences in remission or continuation rate by initial bDMARD agent in either disease. Indeed, there were no significant differences in any clinical outcome measure in RA. In PsA, patients on etanercept at baseline also had a lower CRP at 12 years (*p* = 0.041). However, given the normal values in both groups, this is of dubious significance.

Strengths of this study include the large cohort of real-world patients with detailed clinical, laboratory and radiological outcomes. We are not aware of any study of IA outcomes with bDMARD therapy with such a long follow-up period. We highlight important demographic and outcome differences between RA and PsA and identify predictors of both long-term remission and bDMARD continuation. We compare 12-year outcomes for adalimumab and etanercept. An important comparison given these two agents still account for the majority of bDMARD prescriptions for IA in Ireland [32]. One limitation of this study is the patients lost to follow-up, which is inevitable in a real-world study of patients. Another limitation is that DAS28-CRP defined remission does not account for important extra-articular manifestations of psoriatic arthritis, such as the skin, nail, entheseal and spinal involvement, although this was not an aim of this study.

## Conclusion

In conclusion, a greater percentage of PsA patients achieves remission on bDMARD therapy compared to RA patients. Both achieve higher frequencies of remission compared to previous analyses of csDMARD therapy alone. PsA patients had significantly higher levels of employment and education. One-year bDMARD persistence and low-disease activity at 1 year were predictive of a 12-year persistence. Male gender and 12-year persistence predicted a 12-year remission. In a comparison of adalimumab and etanercept, outcomes were very well matched with no differences in remission or continuation rate in either RA or PsA.

## Supplementary Information

**Additional file 1: Table S1.** Baseline demographics of RA patients on subcutaneous biologics. **Table S2.** Baseline demographics of PsA patients on subcutaneous biologics. **Table S3.** Clinical outcomes of RA patients by initial subcutaneous biologic. **Table S4.** Clinical outcomes of PsA patients by initial subcutaneous biologic.

## Data Availability

The datasets used and analysed during the current study are available from the corresponding author on reasonable request.
