# Supplementary material for: Long-term remission and biologic persistence rates: 12-year real-world data
Source: Arthritis Res Ther. 2021 Jan 13;23:25. doi: 10.1186/s13075-020-02380-z (PMC7807520; doi:10.1186/s13075-020-02380-z)
Supplement: Supplementary file 1 — Additional file 1: Table S1. Baseline demographics of RA patients on subcutaneous biologics. Table S2. Baseline demographics of PsA patients on subcutaneous biologics. Table S3. Clinical outcomes of RA patients by initial subcutaneous biologic. Table S4. Clinical outcomes of PsA patients by initial subcutaneous biologic. [file 13075_2020_2380_MOESM1_ESM.docx]

**Supplementary Table 1. Baseline demographics of RA patients on subcutaneous biologics**

|  | **Adalimumab (n=144)** | **Etanercept**  **(n=100)** | **p** |
| --- | --- | --- | --- |
| **Age**, years | 56.6 (11.2) | 52.9 (12.7) | 0.019 |
| **Female** | 206 (73.6%) | 76 (76.0%) | NS |
| **Disease Duration**, years | 10 (0-51) | 8.5 (0-40) | NS |
| **Educational level**  Primary School  Secondary  University | 128 (100%)  94 (73.4%)  29 (22.7%) | 95 (100%)  69 (72.6%)  21 (22.1%) | NS |
| **Smoking status**  Never  Ex  Current | 55 (41.7%)  48 (36.4%)  29 (22.0%) | 29 (30.9%)  35 (37.2%)  30 (31.9%) | NS |
| **Hours worked**, weekly | 37.1 (10.9) | 38.3 (13.3) | NS |
| **Employment**  Employed  Unemployed  Student  Other | 22 (66.7%)  5 (15.2%)  0  6 (18.2%) | 14 (66.7%)  3 (14.3%)  0  4 (19.0%) | NS |
| **Methotrexate** | 95 (66%) | 65 (65%) | NS |
| **HAQ score** | 1.26 (0.64) | 1.29 (0.65) | NS |
| **EMS**, minutes | 30 (0-1440) | 30 (0-1440) | NS |
| **RF positive** | 103 (76.3%) | 73 (79.3%) | NS |
| **ACPA positive** | 20 (80%) | 10 (83.3%) | NS |
| **Erosions*** | 46 (46.5%) | 35 (50.0%) | NS |

Values are expressed as n (%), mean (SD) or median (range). HAQ…Health Assessment Questionnaire, EMS…early morning stiffness, RF…Rheumatoid factor, Anti-CCP…anti-cyclic citrullinated peptide, *On X-rays of hands and feet.

**Supplementary Table 2. Baseline demographics of PsA patients on subcutaneous biologics**

|  | **Etanercept**  **(n=68)** | **Adalimumab (n=47)** | **p** |
| --- | --- | --- | --- |
| **Age** | 44.1 (11.7) | 45.0 (12.0) | NS |
| **Female** | 40 (58.8%) | 25 (53.2%) | NS |
| **Disease Duration,** years | 7 (0-34) | 8 (0-40) | NS |
| **Educational level**  Primary School  Secondary  University | 38 (100%)  46 (88.5%)  28 (53.8%) | 52 (100%)  37 (97.4%)  26 (68.3%) | NS |
| **Smoking status**  Never  Ex  Current | 15 (83.3%)  1 (5.6%)  1 (5.6%) | 15 (100%)  0  0 | NS |
| **Hours worked**, weekly | 42.7 (9.9) | 39.6 (8.5) | NS |
| **Employment**  Employed  Unemployed  Student | 34 (54.0%)  18 (28.6%)  11 (17.5%) | 12 (31.6%)  15 (39.5%)  11 (28.9%) | 0.029  NS  NS |
| **Methotrexate** | 15 (22.1%) | 16 (34.8%) | NS |
| **HAQ score** | 0.94 (0.66) | 0.88 (0.62) | NS |
| **EMS**, minutes | 30 (0-1440) | 45 (0-1440) | NS |
| **Erosions*** | 15 (30.6%) | 9 (32.1%) | NS |

Values are expressed as n (%), mean (SD) or median (range). HAQ…Health Assessment Questionnaire, EMS…early morning stiffness, *on X-rays of hands and feet

**Supplementary Table 3. Clinical outcomes of RA patients by initial subcutaneous biologic**

|  | **Baseline** | | | **1 year** | | | **12 years** | | |
| --- | --- | --- | --- | --- | --- | --- | --- | --- | --- |
|  | ADA  (n=144) | ETA  (n=100) | p | ADA  (n=104) | ETA  (n=74) | p | ADA  (n=99) | ETA  (n=63) | p |
| Persistence | n/a | n/a | n/a | 56  (56%) | 67  (47%) | NS | 28  (49%) | 34 (35%) | NS |
| Methotrexate | 65 (65%) | 95  (66%) | NS | 42  (66%) | 51  (61%) | NS | 27  (47%) | 39 (42%) | NS |
| PGH | 60  (0-100) | 60  (0-100) | NS | 30  (0-100) | 40  (0-100) | NS | 40  (0-100) | 40  (0-100) | NS |
| TJC28 | 9 (0-28) | 9 (0-28) | NS | 1 (0-19) | 1 (0-16) | NS | 0 (0-19) | 0 (0-15) | NS |
| SJC28 | 9  (0-25) | 10  (0-24) | NS | 1  (0-21) | 1  (0-16) | NS | 0  (0-15) | 0  (0-15) | NS |
| CRP | 18  (4-155) | 16  (2-100) | NS | 4  (1-60) | 4  (1-36) | NS | 3.15  (1-53) | 3.2  (1-42) | NS |
| DAS28-CRP | 5.26 (1.17) | 5.26 (1.04) | NS | 3.15 (1.14) | 3.21 (1.16) | NS | 2.48 (1.03) | 2.42 (0.90) | NS |
| Remission | 2  (2%) | 0 | NS | 21  (35%) | 31  (35%) | NS | 23  (59%) | 37 (66%) | NS |
| Progression of erosions | | | | 20  (30%) | 14  (30%) | NS | 28  (33%) | 20 (34%) | NS |

ADA…Adalimumab, ETA…Etanercept, PGH…Patient Global Health Visual Analogue Score (mm), TJC28…28-joint tender joint count, SJ28…28-joint swollen joint count

**Supplementary Table 4. Clinical outcomes of PsA patients by initial subcutaneous biologic**

|  | **Baseline** | | | **1 year** | | | **12 years** | | |
| --- | --- | --- | --- | --- | --- | --- | --- | --- | --- |
|  | ETA  (n=68) | ADA  (n=47) | p | ETA  (n=51) | ADA  (n=32) | p | ETA  (n=49) | ADA  (n=27) | p |
| Persistence | n/a | n/a | n/a | 43 (63%) | 23 (49%) | NS | 26 (54%) | 14 (52%) | NS |
| Methotrexate | 15 (22%) | 16 (35%) | NS | 8 (17%) | 9  (35%) | NS | 9 (18%) | 9 (36%) | NS |
| PGH* | 50  (0-100) | 50  (0-100) | NS | 20  (0-80) | 20  (0-90) | NS | 7  (0-100) | 44  (0-55) | NS |
| TJC28 | 8  (0-28) | 5  (0-17) | NS | 0  (0-20) | 0  (0-9) | NS | 0  (0-22) | 0  (0-3) | NS |
| SJC28 | 6  (0-22) | 4  (0-24) | NS | 0  (0-25) | 0  (0-5) | NS | 0  (0-1) | 0  (0-2) | NS |
| CRP | 10  (4-86) | 10  (0-108) | NS | 4  (0-41) | 4  (1-17) | NS | 1.1  (1-46) | 2.1  (1-31) | 0.041 |
| DAS28-CRP | 4.95 (1.10) | 4.45 (0.93) | 0.011 | 2.47 (1.26) | 2.51 (0.94) | NS | 1.60 (0.71) | 2.23 (0.58) | NS |
| Remission | 1 (2%) | 0 | NS | 26 (64%) | 14 (61%) | NS | 17 (94%) | 2 (67%) | NS |
| Progression of erosions | | | | 2 (5.4%) | 3  (20%) | NS | 6 (13%) | 4 (16%) | NS |

ADA…Adalimumab, ETA…Etanercept, PGH…Patient Global Health Visual Analogue Score (mm), TJC28…28-joint tender joint count, SJ28…28-joint swollen joint count
